# Revealing a Novel Landscape of the Association Between Blood Lipid Levels and Alzheimer's Disease: A Meta-Analysis of a Case-Control Study

**DOI:** 10.3389/fnagi.2019.00370

**Published:** 2020-02-05

**Authors:** Qianyun Tang, Fengling Wang, Jingjing Yang, Hua Peng, Yu Li, Bin Li, Shuhong Wang

**Affiliations:** ^1^Xiangya Hospital, Central South University, Changsha, China; ^2^Xiangya School of Nursing, Central South University, Changsha, China; ^3^Department of Geriatrics, Xiangya Hospital, Central South University, Changsha, China; ^4^Department of Neurology, Xiangya Hospital, Central South University, Changsha, China; ^5^National Clinical Research Center for Geriatric Disorders, Geriatric Department, Xiangya Hospital, Central South University, Changsha, China

**Keywords:** Alzheimer's disease, blood lipid levels, total cholesterols, high-density lipoprotein cholesterol, low-density lipoprotein cholesterol, triglyceride, biomarker, meta-analysis

## Abstract

**Objectives:** Blood lipid profiles have been ambiguously reported as biomarkers of AD in recent years. This study was conducted to evaluate the correlation between blood lipid levels and AD in later-life and to explore the effectiveness and reliability of blood lipid profiles as biomarkers of AD.

**Methods:** Database searching was conducted using PubMed, the Cochrane Library, EMBASE, and Medline. This study was designed following the Meta-analysis of Observational Studies in Epidemiology (MOOSE) criteria. Review Manager 5.3 (RevMan 5.3) software was adopted to perform meta-analysis evaluating the standard mean difference (SMD) with its 95% confidence intervals (CI).

**Results:** A total of 5,286 participants were enrolled from 27 case–control studies in this meta-analysis. The pooled results demonstrated that total cholesterol (TC) level was significantly associated with AD in late-life (SMD = 0.17, 95% CI: [0.01, 0.32], *P* = 0.03), especially in the subgroup under 70 years old (SMD: 0.45, 95% CI: [0.11, 0.79], *P* = 0.01) and the subgroup of Western population (SMD: 0.29, 95% CI: [0.04, 0.53], *P* = 0.02). In the subgroup under 70 years old, the high-density lipoprotein cholesterol (HDL-C) level (SMD = −0.50, 95% CI: [−0.76, −0.25], *P* = 0.0001) and the low-density lipoprotein cholesterol (LDL-C) level (SMD = 0.59, 95% CI: [0.02, 1.16], *P* = 0.04) in the AD group were significantly lower and higher than in the control group, respectively. In the subgroup with a sample size larger than 100 subjects, the LDL-C level was significantly higher in AD patients than in the control elderly group (SMD = 0.31, 95% CI: [0.05, 0.56], *P* = 0.02). There was no significant association between triglyceride (TG) levels and AD in later-life (SMD = −0.00, 95% CI: [−0.12, 0.12], *P* = 1.00).

**Conclusion:** TC can be a new predictive biomarker of AD or cognitive decline in later-life. Increased TC levels are found to be associated with an elevated risk of AD. Decreased HDL-C levels and increased LDL-C levels may relate to an elevated risk of AD in subjects aged 60–70. Further comprehensive researches will be necessary in the future.

## Introduction

Alzheimer's Disease (AD) is the most common type of all neurodegenerative diseases and is marked with three continuous stages, which includes preclinical stage, mild cognitive impairment (MCI) stage, and dementia stage (Zarrouk et al., 2018). Around 50 million people are suffering from dementia worldwide, two-thirds of whom are diagnosed with AD. The brain of an AD patient is identified as synaptic dysfunction, tau phosphorylation and accumulation of the amyloid-β peptide (Aβ) in brain tissues (Querfurth and LaFerla, 2010); the 42-residue form of Aβ (Aβ42) is the main segment of the deposits (Sittiwet et al., 2018). AD kills more people in the U.S. than breast cancer and prostate cancer combined. It has developed into a major public health problem and there will be a new case somewhere in the world every 3 s (Patterson, 2018).

In the past, behavioral testing and neuroimaging were commonly used to diagnose AD. The concentration of Aβ42 and the phosphorylation of total-tau (t-tau) and phospho-tau (p-tau) (Hampel et al., 2011; Dubois et al., 2014) are accepted as biomarkers of AD (Zarrouk et al., 2018). However, these methods may not only be invasive but also unaffordable to some patients. Blood lipid profiles as biomarkers may be more accessible, affordable, and less invasive modalities to detect and diagnose AD (Zarrouk et al., 2018; Liu et al., 2019). Some lipids extracted from peripheral blood have been validated to predict development to either amnestic mild cognitive impairment (aMCI) or AD with over 90% accuracy (Mapstone et al., 2014). The blood-based lipid profiles were an appropriate source for AD biomarker screening (Zarrouk et al., 2018).

Alois Alzheimer noted that there are cholesterol and triglycerides inside lipid droplets, the accumulation of which was related to cellular stress and resulting in AD (Alzheimer, 1907). Blood lipid profiles have potentially modifiable influencing factors, including age, sex, diet, exercise, medications, educational levels, and/or lifestyle, such as smoking, eating habits, and various other personal choices. Measures can be applied earlier to prevent worsening cognitive decline potentially when blood lipid levels are abnormal. Studies on the risk factors of AD are critical to the prevention and treatment of AD and cognitive impairment. Nevertheless, there is still no coherent conclusion in this field. The parameters of blood lipid profiles have been recognized as potential biomarkers and have been reported to be associated with the risk of AD, which include total cholesterol (TC), high-density lipoprotein cholesterol (HDL-C), low-density lipoprotein cholesterol (LDL-C), and triglyceride (TG) (Wu et al., 2019). Lipids including TC and HDL-C are currently used as tools to assess the risk of AD and dementia in midlife (Anstey et al., 2017). It has been proven that middle-aged high cholesterol levels are related to cognitive impairment or AD in old age (Rantanen et al., 2017), and midlife cholesterol has been shown to predict AD (Kivipelto et al., 2001; Strand et al., 2013). However, the relationship between AD and cholesterol or lipid levels in the elderly has not been determined yet.

The opinion that blood lipid profiles can be biomarkers of AD is still ambiguous, which is limited by a lack of compatible data for pooling on the association between lipid and cognitive outcomes (Pappolla et al., 2003; Popp et al., 2012). The present study aims to evaluate the link between blood lipid levels and AD and whether they are reliable biomarkers of AD in the later-life of patients.

## Methods

### Search Strategy

This study was designed following the Meta-analysis of Observational Studies in Epidemiology (MOOSE) criteria (Stroup et al., 2000; Moher et al., 2009). Databases including PubMed, Embase, the Cochrane Library and Medline were retrieved for qualified literature that was published before March 2019. Only English-language literatures were reviewed in this study. The search strategy followed the PICOS principles. Mesh terms and topic terms were used as the searching term, including “Cholesterol,” and “Alzheimer's disease,” “AD,” “Alzheimer^*^,” “Senile Dementia,” “Alzheimer Type Dementia,” “Alzheimer-Type Dementia,” and “ATD.” Additional published literature identified in the selection of the reference of the initially retrieved articles was also investigated in our meta-analysis.

### Inclusion Criteria

Studies were identified eligible if they met the criteria as defined below:

1. Patients who were diagnosed with AD and at least 60 years old were defined as participants of the case group. The diagnosis criteria were based on the National Institute of Neurological and Communicative Disorders and Stroke and the Alzheimer's Disease and Related Disorders Association (NINCDS-ADRDA) (McKhann et al., 1984).
2. Participants did not use lipid-lowering drugs during the study.
3. The mean age of the population was older than 60 years old.
4. The control group was composed of the counterpart of the case group who were not diagnosed with AD and had normal cognition.
5. The study reported its original data in the mean, standard deviation (SD), or standard errors (SE) of blood lipid profiles (TC, HDL-C, LDL-C, and TG).
6. Case-control studies of human beings are eligible for this review.
7. Studies were published in English between January 2000 and March 2019.

### Exclusion Criteria

1. Twin studies and studies with duplicate data, incomplete or erroneous reporting of data.
2. Case-control studies reporting other types of dementia such as vascular dementia, dementia with Lewy bodies, frontotemporal dementia, mixed dementia and other secondary or post-traumatic dementia, not AD.
3. The participants of the control group were diagnosed with other types of dementia such as vascular dementia, dementia with Lewy bodies, frontotemporal dementia, mixed dementia, and other secondary or post-traumatic dementia.
4. Participants who were suffering from severe physical or mental disease.
5. The quality of literature judged by the Newcastle-Ottawa Scale was low (scores < 5).
6. Studies reporting inadequate information, for example, data only contained mean without SD/SE.

### Data Extraction

The following data have been extracted independently and cross-checked by two authors (Qianyun Tang and Fengling Wang): first authors, titles of references, time of publication, nations, and regions, study designs, sample sizes, data sources, mean ages, outcomes, quality scores, and diagnostic criteria of AD. The extracted data were noted in an Excel file, which was checked by the third author (Shuhong Wang). Any disagreements were resolved after discussion.

### Quality Evaluation

Studies were assessed for bias using the Newcastle–Ottawa Scale (NOS) by two reviewers (Qianyun Tang and Fengling Wang) (Stang, 2010). There will be a third reviewer (Shuhong Wang) to discuss the results if any disagreement exists. Each case-control study was evaluated according to eight points covering three parts as follows: selection, comparability, and exposure. We use stars to symbolize scores of each item in the scale, with one star representing one point and nine stars as the highest quality. Studies with scores <5 were regarded as low quality.

### Statistical Analysis

Review Manager 5.3 (RevMan 5.3) software was applied to conduct the present meta-analysis. Dichotomous data were reported in terms of the odds ratio (OR), with 95% CI, and continuous data were reported in terms of the standard mean difference (SMD), with 95% CI. An α value equal to 0.1 and a *P*-value of < 0.05 were considered statistically significant. The heterogeneity of each study was analyzed using the *I*^2^ statistic and the Cochrane-Q test. Studies with an *I*^2^ statistic between 25 and 50% were considered to be low-heterogeneous, those between 50 and 75% to be moderate-heterogeneous, and those greater than 75% to be high-heterogeneous. In a study with a *P* > 0.1, or *I*^2^ ≤ 50%, signifying little heterogeneity between studies, a fixed-effect model was employed, or a random effect model was adopted.

## Results

### Search Results

**Figure 1:**
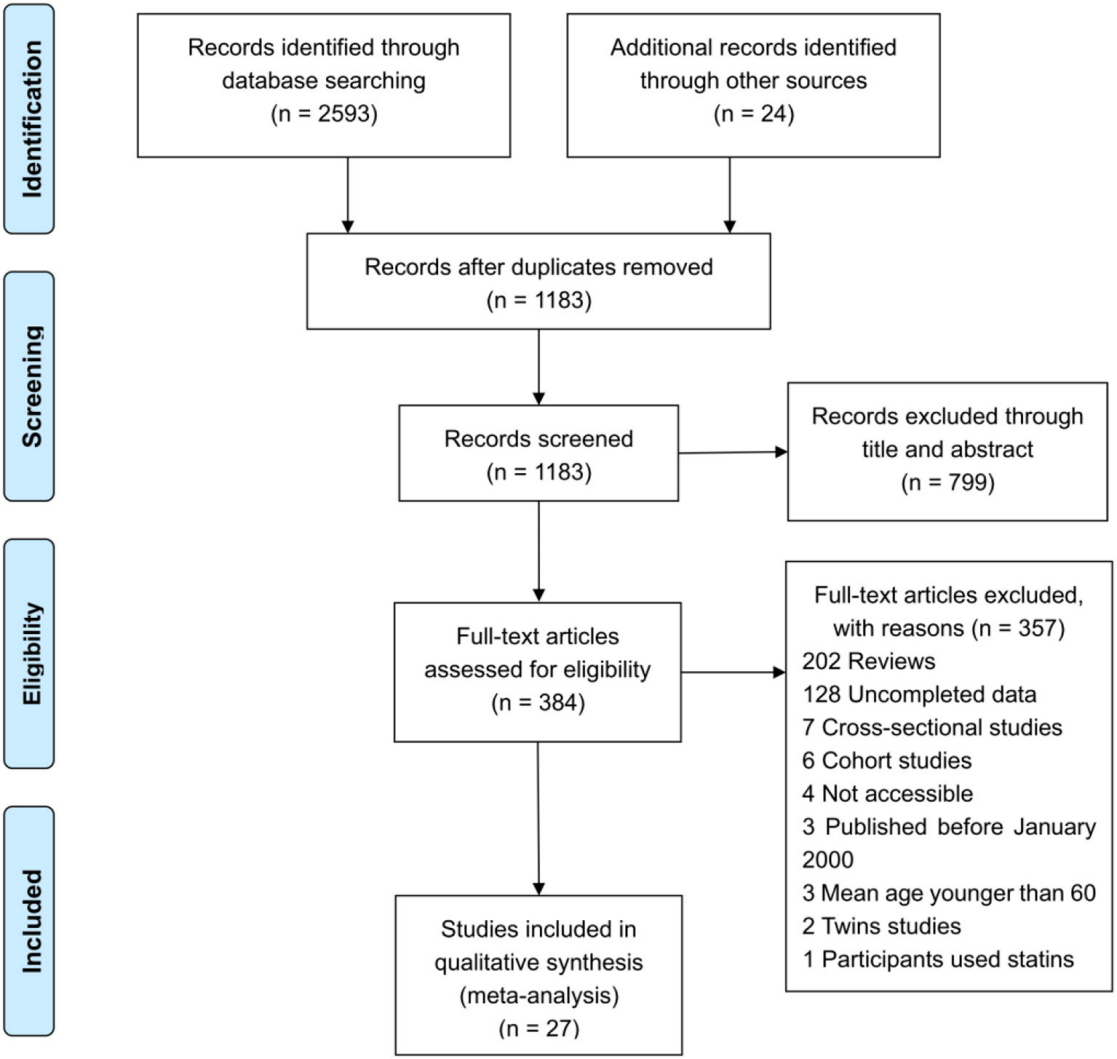
Flow diagram of reference selection process.

The literature search yielded a total of 2,617 articles, with 2,593 articles collected through database searching and 24 additional articles identified through other searching methods. A total of 1,183 articles remained after duplicate articles were removed. After reviewing the titles and abstracts, 799 articles were found to be unrelated and then excluded, while 384 studies were identified as potentially eligible for inclusion. After reviewing the full article, 357 articles were excluded, since 232 were reviews, 35 were cross-sectional studies, 22 were reporting incomplete data, 18 were cohort studies, 15 involved subjects whose mean age was under 60, 15 were animal trials, 10 were inaccessible, 8 were twin studies, and 2 had participants using statins. After excluding the above papers, 27 articles (Table 1) were eligible for inclusion in this meta-analysis (de Bustos et al., 2000; Wada, 2000; Hoshino et al., 2002; Schonknecht et al., 2002; Borroni et al., 2003; Teunissen et al., 2003; Watanabe et al., 2004; Cankurtaran et al., 2005; Yamamoto et al., 2005; Raygani et al., 2006; Ban et al., 2009; Cascalheira et al., 2009; Kolsch et al., 2010; Zengi et al., 2011; Popp et al., 2012, 2013; Singh et al., 2012; Xiao et al., 2012; Alam et al., 2014; Chang et al., 2014; Zhao et al., 2014; Li et al., 2015b, 2017; Yassine et al., 2016; Zheng et al., 2016; Kouzuki et al., 2018; Chen et al., 2019). One Flow diagram of the literature selection is provided in Figure 1. The results of the quality assessment are displayed in Table 2.

**Table 1:** Characteristics of studies included in the meta-analysis.

**References**	**Nation**	**Region**	**Study type**	**Data source**	**Sample size**		**Mean age (range)**		**Gendar ratio (M/F)**		**Outcome**	**NOS scores**
					**AD**	**Control**	**AD**	**Control**	**AD**	**Control**		
Chen et al. (2019)	China	Asia	Case-control	Single-centered	117	117	67.64 ± 6.65	66.06 ± 6.00	56/61	44/73	TC, TG HDL-C, LDL-C	7
Kouzuki et al. (2018)	Japan	Asia	Case-control	Single-centered	42	18	80.5 ± 5.7	75.6 ± 5.5	16/26	5/13	TC, TG HDL-C, LDL-C	5
Li et al. (2017)	China	Asia	Case-control	Single-centered	118	120	71.81 ± 9.66	70.45 ± 9.52	75/43	68/52	TC, TG HDL-C, LDL-C	6
Zheng et al. (2016)	China	Asia	Case-control	Multi-centered	207	256	80.67 ± 8.19	81.66 ± 6.38	68/139	89/167	TC, TG HDL-C, LDL-C	5
Yassine et al. (2016)	America	North America	Case-control	Single-centered	26	47	77 ± 10	78 ± 7	9/17	20/27	TC	7
Alam et al. (2014)	India	Asia	Case-control	Single-centered	75	120	66.2 ± 9.2	63.8 ± 8.2	na	na	TC, TG HDL-C, LDL-C	8
Chang et al. (2014)	China	Asia	Case-control	Multi-centered	44	62	80 ±8.92	79.63 ± 7.85	na	na	TC, TG, HDL-C	6
Li et al. (2015a)	China	Asia	Case-control	Single-centered	201	257	76.79 ± 5.65	75.88 ± 6.50	90/111	121/136	TC, TG HDL-C, LDL-C	6
Zhao et al. (2014)	China	Asia	Case-control	Single-centered	48	37	69.32 ± 5.53	71.06 ± 5.87	23/25	19/18	TC, TG HDL-C, LDL-C	5
Popp et al. (2013)	Switzerland	Europe	Case-control	Single-centered	106	87	71.1 ± 7.87	67.7 ± 9.13	36/68	44/43	TC	8
Xiao et al. (2012)	China	Asia	Case-control	Single-centered	104	104	77.8 ± 6.74	76.5 ± 6.14	57/47	56/48	TC, TG HDL-C, LDL-C	5
Popp et al. (2012)	Switzerland	Europe	Case-control	Single-centered	53	43	71.23 ± 8.29	67.33 ± 9.04	20/33	21/22	TC	8
Zengi et al. (2011)	Turkey	Asia	Case-control	Single-centered	21	20	76 ± 7.8	81 ± 7.2	10/11	11/9	TC, TG HDL-C, LDL-C	7
Singh et al. (2012)	India	Asia	Case-control	Single-centered	70	75	50-85	50-85	na	na	TC, TG HDL-C, LDL-C	6
Kolsch et al. (2010)	Germany	Europe	Case-control	Multi-centered	411	201	71.9 ± 8.5	69.5 ± 6.9	162/249	106/95	TC	5
Cascalheira et al. (2009)	Portugal	Europe	Case-control	Single-centered	19	36	75.6 ± 2.11	70.7 ± 1.73	10/9	18/18	TC	8
Ban et al. (2009)	Japan	Asia	Case-control	Single-centered	197	47	80 ± 14.04	75 ± 6.86	79/118	29/18	TG, HDL-C, LDL-C	5
Raygani et al. (2006)	Iran	Asia	Case-control	Multi-centered	94	111	74.2 ± 10	72 ± 11.4	41/53	41/70	TC, TG HDL-C, LDL-C	7
Cankurtaran et al. (2005)	Turkey	Asia	Case-control	Single-centered	120	803	74 ± 7.6	71.4 ± 5.9	41/79	297/504	TC, TG HDL-C, LDL-C	7
Yamamoto et al. (2005)	Japan	Asia	Case-control	Single-centered	61	32	80 ± 6	77 ± 5	24/37	17/15	TC, TG HDL-C, LDL-C	6
Watanabe et al. (2004)	Japan	Asia	Case-control	Single-centered	34	63	76 ± 9	72 ± 11	34 man only	63 man only	TC, TG HDL-C, LDL-C	6
Borroni et al. (2003)	Italy	Europe	Case-control	Single-centered	60	45	71.4 ± 9.7	71.2 ± 8.7	22/38	16/29	TC	7
Teunissen et al. (2003)	The Netherlands	Europe	Case-control	Single-centered	34	61	73.22 ± 10.04	68.39 ± 6.68	14/20	35/26	TC	6
Schonknecht et al. (2002)	Germany	Europe	Case-control	Single-centered	14	10	75.4 ± 10.3	69.0 ± 5.8	8/6	6/4	TC	7
Hoshino et al. (2002)	Japan	Asia	Case-control	Single-centered	82	40	77 ± 6.8	84.2 ± 3.1	23/59	13/27	HDL-C, LDL-C	5
Wada (2000)	Japan	Asia	Case-control	Single-centered	36	15	77.3 ± 4.9	71.8 ± 6.1	17/19	2/13	TC, TG HDL-C, LDL-C	6
de Bustos et al. (2000)	Spain	Europe	Case-control	Multi-centered	44	21	73.8 ± 8.3	69.3 ± 5.6	18/26	8/13	TC	7

*AD, Alzheimer's Disease; TC, Total Cholesterol; TG, Triglyceride; HDL-C, High-density Lipoprotein Cholesterol; LDL-C, Low-density Lipoprotein Cholesterol; M, male; F, female; MMSE, Mini-Mental State Examination; NOS, Newcastle-Ottawa Scale; na, not available*.

**Table 2:** Newcastle-Ottawa quality assessment scale for case control studies.

**References**	**Selection**				**Comparability**	**Exposure**			**Quality Scores♮**
	**Definition of cases**	**Representativeness of cases**	**Selection of Controls**	**Definition of Controls**	**Basis of the design or analysis**	**Ascertainment of exposure**	**Same method of ascertainment for cases and controls**	**Non-Response rate**	
Chen et al. (2019)	⋆	⋆	⋆	⋆	⋆⋆	-	⋆	-	7
Kouzuki et al. (2018)	⋆	-	⋆	⋆	⋆	-	⋆	-	5
Li et al. (2017)	⋆	⋆	-	⋆	⋆⋆	-	⋆	-	6
Zheng et al. (2016)	⋆	⋆	-	⋆	⋆	-	⋆	-	5
Yassine et al. (2016)	⋆	⋆	⋆	⋆	⋆	⋆	⋆	-	7
Alam et al. (2014)	⋆	⋆	⋆	⋆	⋆⋆	⋆	⋆	-	8
Chang et al. (2014)	⋆	⋆	-	⋆	⋆	⋆	⋆	-	6
Li et al. (2015a)	⋆	-	⋆	⋆	⋆	⋆	⋆	-	6
Zhao et al. (2014)	⋆	-	-	⋆	⋆	⋆	⋆	-	5
Popp et al. (2013)	⋆	⋆	⋆	⋆	⋆⋆	⋆	⋆	-	8
Xiao et al. (2012)	⋆	-	-	⋆	⋆	⋆	⋆	-	5
Popp et al. (2012)	⋆	⋆	⋆	⋆	⋆⋆	⋆	⋆	-	8
Zengi et al. (2011)	⋆	⋆	⋆	⋆	⋆	⋆	⋆	-	7
Singh et al. (2012)	⋆	⋆	⋆	⋆	⋆	-	⋆	-	6
Kolsch et al. (2010)	⋆	-	-	⋆	⋆	⋆	⋆	-	5
Cascalheira et al. (2009)	⋆	⋆	⋆	⋆	⋆⋆	⋆	⋆	-	8
Ban et al. (2009)	⋆	-	⋆	⋆	⋆	-	⋆	-	5
Raygani et al. (2006)	⋆	-	⋆	⋆	⋆⋆	⋆	⋆	-	7
Cankurtaran et al. (2005)	⋆	⋆	-	⋆	⋆⋆	⋆	⋆	-	7
Yamamoto et al. (2005)	⋆	-	⋆	⋆	⋆	⋆	⋆	-	6
Watanabe et al. (2004)	⋆	⋆	⋆	⋆	⋆	-	⋆	-	6
Borroni et al. (2003)	⋆	⋆	⋆	⋆	⋆⋆	-	⋆	-	7
Teunissen et al. (2003)	⋆	-	⋆	⋆	⋆⋆	-	⋆	-	6
Schonknecht et al. (2002)	⋆	-	⋆	⋆	⋆⋆	⋆	⋆	-	7
Hoshino et al. (2002)	⋆	-	⋆	⋆	⋆	-	⋆	-	5
Wada (2000)	⋆	-	⋆	⋆	⋆⋆	-	⋆	-	6
de Bustos et al. (2000)	⋆	⋆	⋆	⋆	⋆⋆	-	⋆	-	7

*Stars are awarded such that the highest-quality studies are awarded up to nine stars. A maximum of one star for each numbered item within the Selection and Exposure categories. A maximum of two stars can be given for Comparability categories. ♮ One star ⋆ for one score*.

### Characteristics of the Included Studies

The characteristics of the 27 included studies are summarized in Table 1. The study designs were all case-control. A total of 5,286 subjects were eligible for the present meta-analysis. All of the studies were published between January 2000 and March 2019. The sample sizes of all studies ranged from 24 to 923. The mean age of the participants of each study ranged from 66 to 80.67 and from 63.8 to 84.2, respectively. The qualities of the collected studies were moderate to good. The NOS scores range 5–8, with an average score of 6.33 (the details are shown in Table 2). Most of the collected studies were single-center trials. In total, 16 studies came from Asia and 9 came from Europe and the Americas (8 came from Europe and 1 came from North America). Diagnostic, inclusion and exclusion criteria for participants were all clearly reported in all the investigated literature. The case groups of those studies were mainly moderate AD according to MMSE scores that have been reported (Folstein et al., 1975). This study mainly evaluated four outcomes: TC, HDL-C, LDL-C, and TG. For further analysis of extreme heterogeneity, we stratified the collected studies into several subgroups.

### Meta-Analysis Results

#### TC and AD

**Figure 2:**
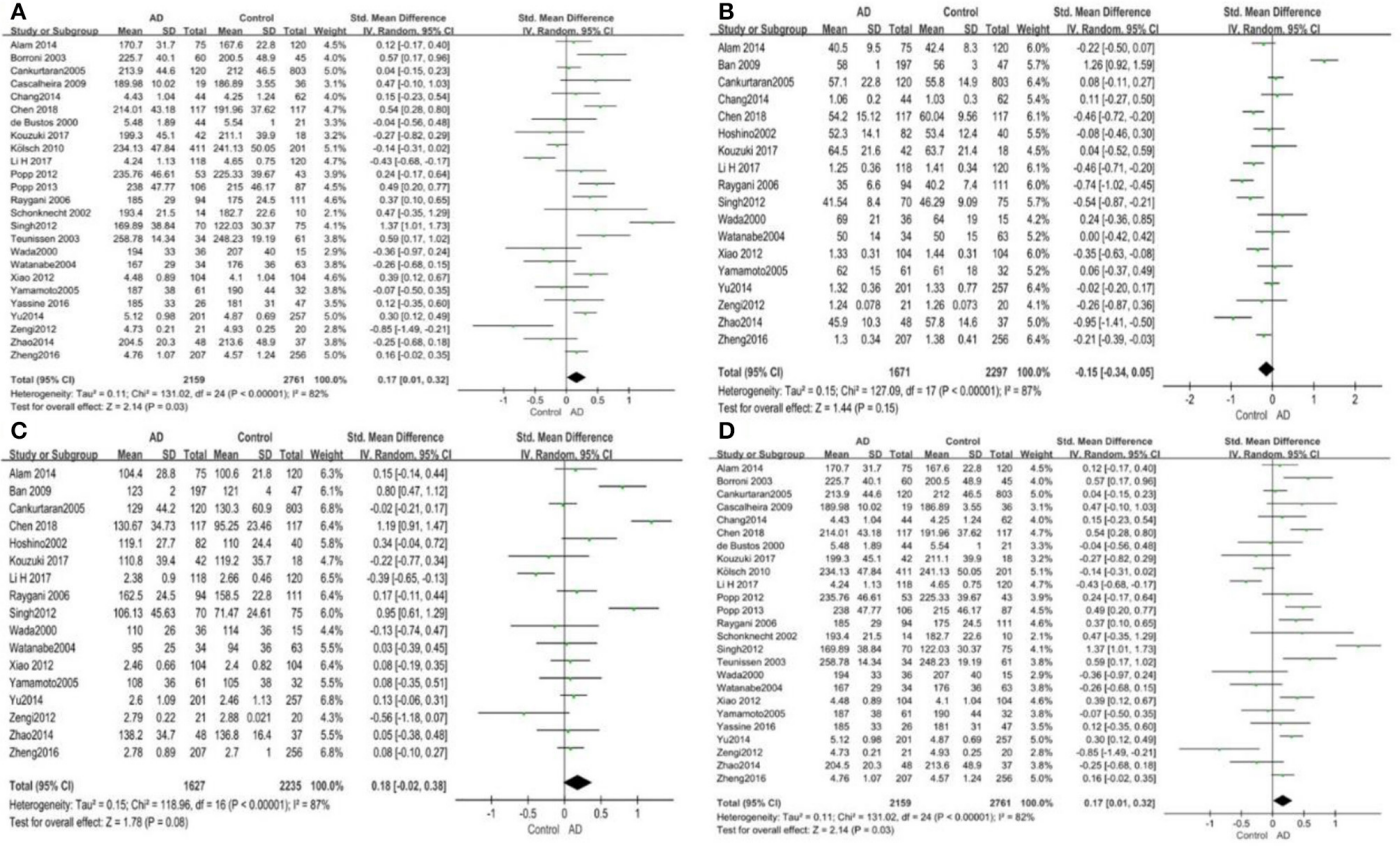
Forest plots of the overall meta-analysis for the effects of the four parameters of blood lipid levels on AD risk with a random-effects model. **(A)** TC levels, **(B)** HDL-C levels, **(C)** LDL-C levels, **(D)** TG levels. SMD, standardized mean difference; CI, confidence interval.

Out of the 27 studies, 25 investigated literature, with a total of 4,920 participants measuring the TC. The results of our study are presented in Table 3. The pooled effects suggested that TC levels in the AD group were significantly higher than in the control group (SMD = 0.17, 95% CI: [0.01, 0.32], *P* = 0.03). Concerning high heterogeneity (*P* < 0.00001; *I*^2^ = 82%), the random-effects model was applied. The forest plot of the pooled analysis is presented in Figure 2A.

**Table 3:** Results of meta-analysis.

**Outcomes of interest**	**Studies, no**.	**AD, no**.	**Control, no**.	**SMD (95%CI)**	*P-*value^*^	**Effect model**	**Heterogeneity**			
							**χ^2^**	**df**	***I*^**2**^, %**	***P*-value**
TC	25	2159	2761	0.17 [0.01, 0.32]	0.03	RE	131.02	24	82	<0.00001
HDL-C	18	1671	2297	−0.15 [−0.34, 0.05]	0.15	RE	127.09	17	87	<0.00001
LDL-C	17	1627	2235	0.18 [−0.02, 0.38]	0.08	RE	118.96	16	87	<0.00001
TG	17	1589	2257	−0.00 [−0.12, 0.12]	1.00	RE	42.09	16	62	0.0004

*AD, Alzheimer's Disease; TC, Total Cholesterol; TG, Triglyceride; HDL-C, High-density Lipoprotein Cholesterol; LDL-C, Low-density Lipoprotein Cholesterol; SMD, standardized mean difference; 95%CI, 95% confidence interval; RE, random effects model*.

**Figure 3:**
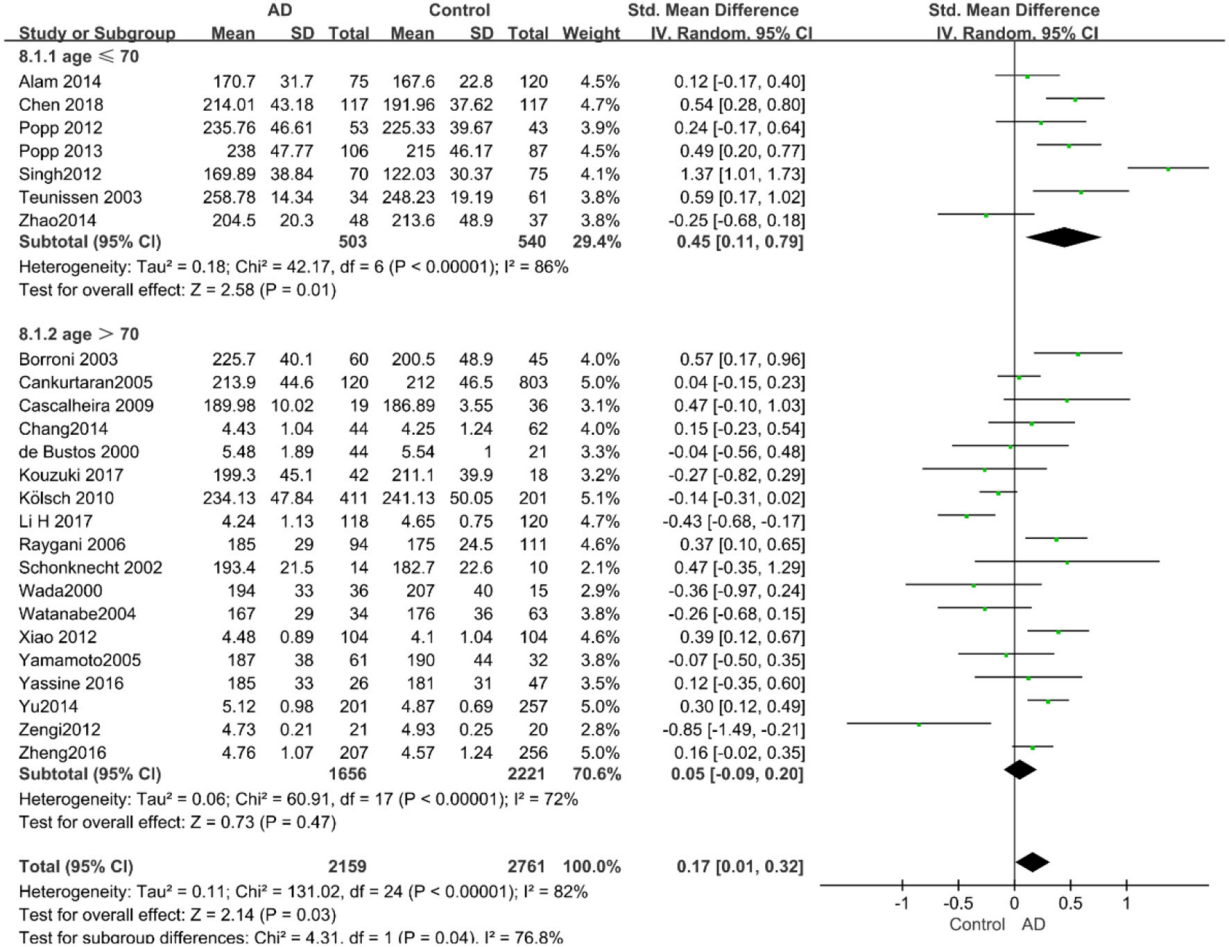
Forest plot of stratified analysis for the effects of TC levels on AD risk in different age groups with a random-effects model. SMD, standardized mean difference; CI, confidence interval.

**Figure 4:**
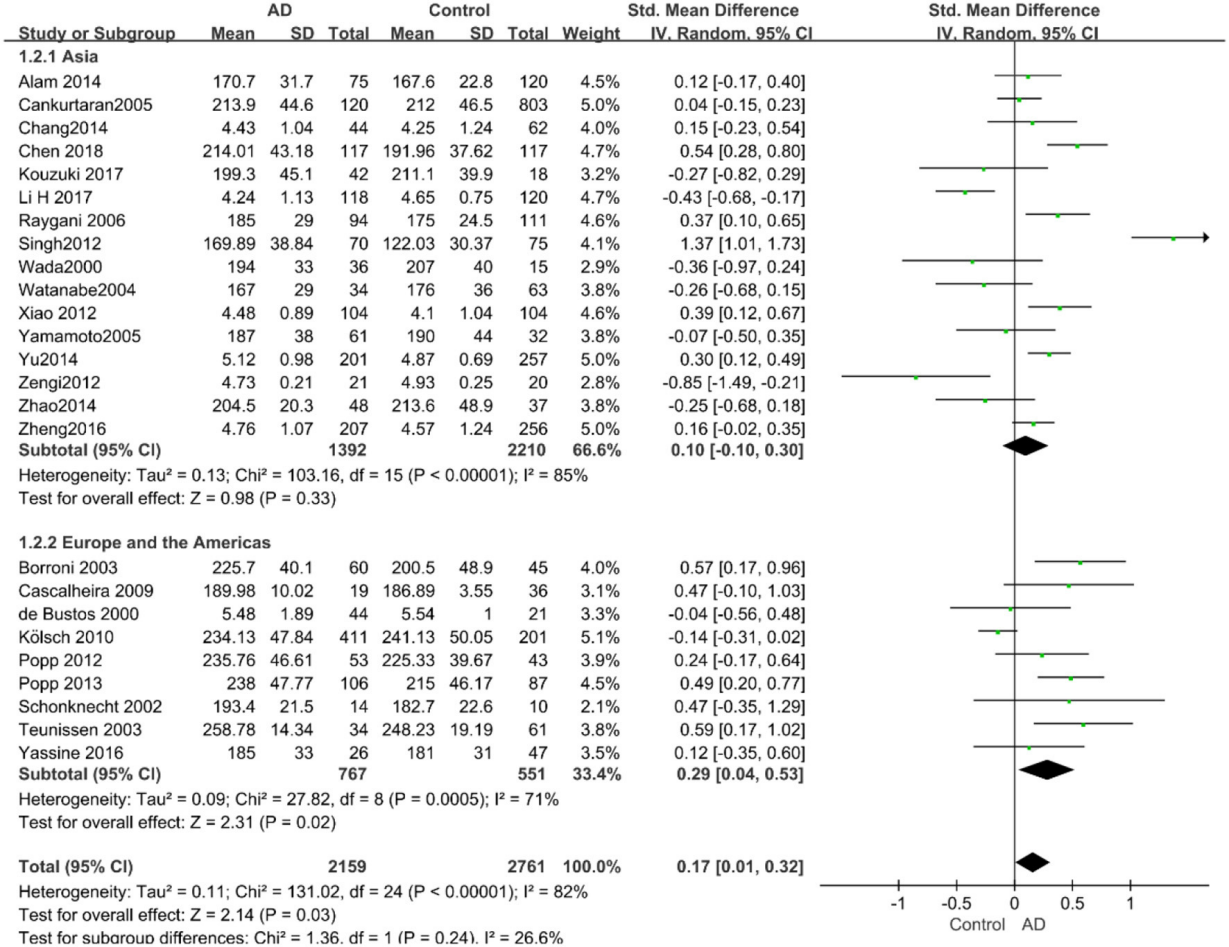
Forest plot of stratified analysis for the effects of TC levels on AD risk in different regions with a random-effects model. SMD, standardized mean difference; CI, confidence interval.

**Figure 5:**
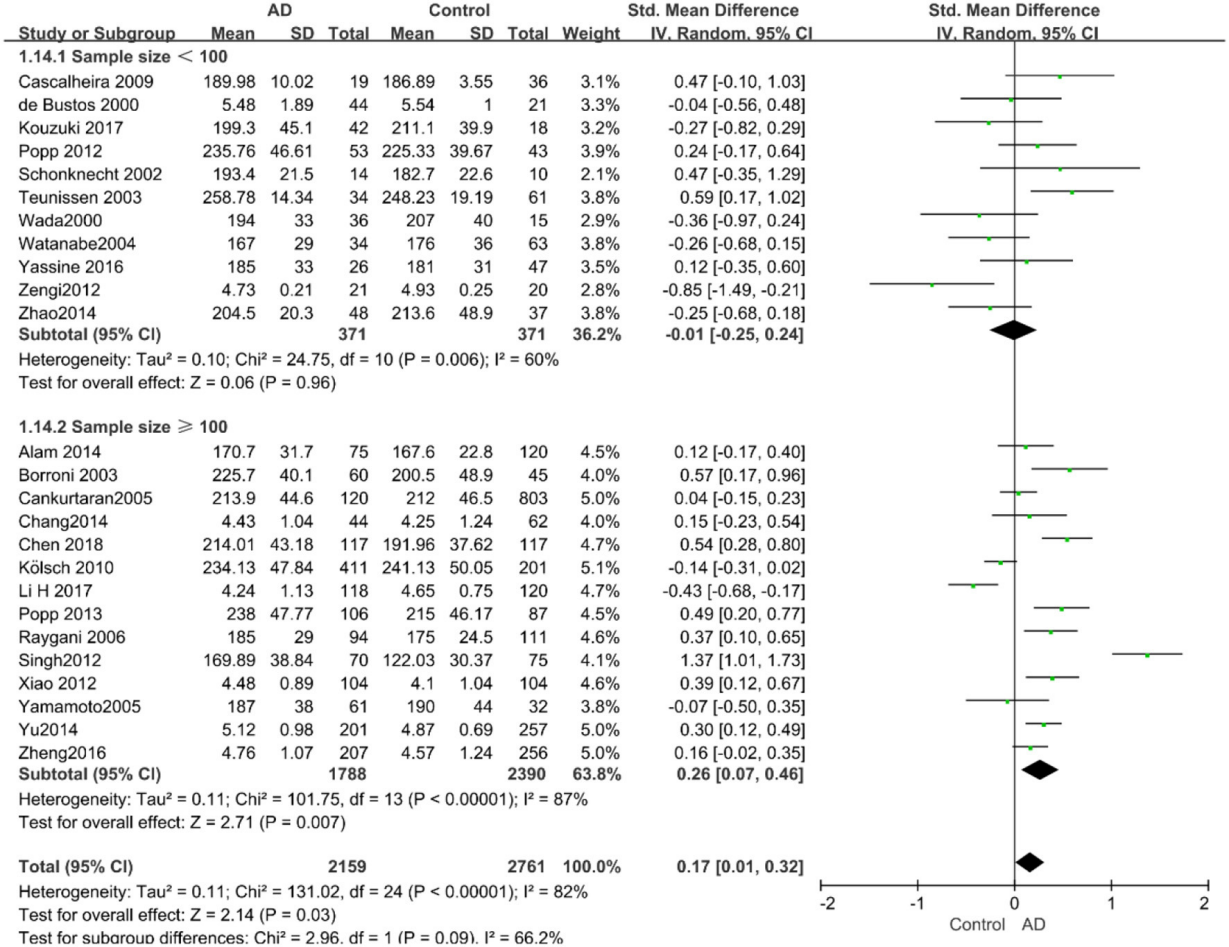
Forest plot of stratified analysis for the effects of TC levels on AD risk in groups with different sample sizes with a random-effects model. SMD, standardized mean difference; CI, confidence interval.

**Figure 6:**
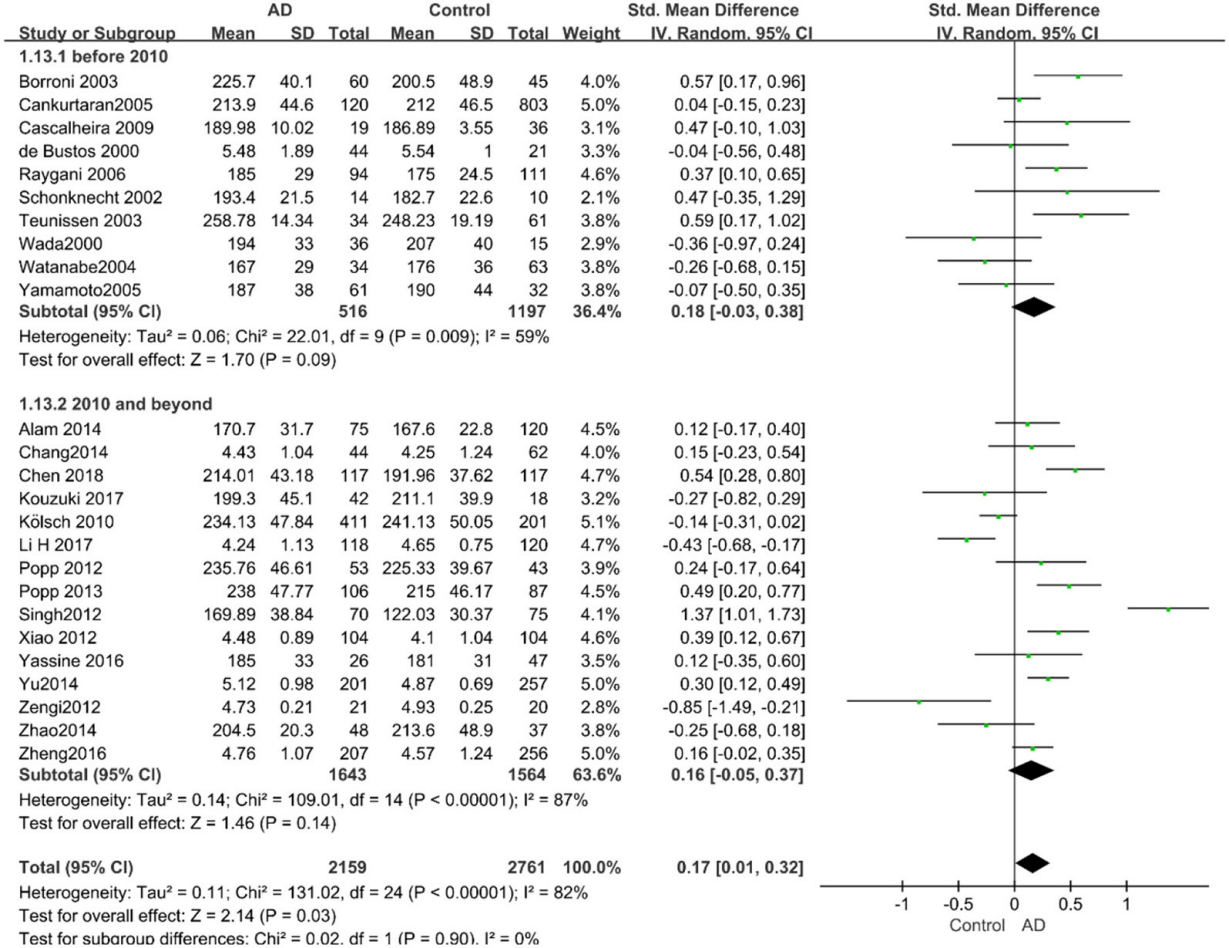
Forest plot of stratified analysis for the effects of TC levels on AD risk in studies published before and after 2010 with a random-effects model. SMD, standardized mean difference; CI, confidence interval.

Given the effects of the mean age of participants, regions of studies, publication time of papers, and sample sizes of studies, we performed a stratified analysis according to these characteristics (the results are presented in Table 4). TC levels were discovered significantly associated with AD in the subgroup of “*mean age* ≤ *70 years old”* (SMD: 0.45, 95% CI: [0.11, 0.79], *P* = 0.01), while no significant difference was found in the subgroup of “*mean age* > *70 years old”* (SMD: 0.05, 95% CI: [−0.09, 0.20], *P* = 0.47; the forest plot is reported in Figure 3). It is inconsistent with the result of a previous study that reported that TC levels had a negative relationship with AD in the population aged 70 years and above (Folstein et al., 1975). No racial or regional differences were found in TC levels between the AD group and the control group in Asia (SMD: 0.10, 95% CI: [−0.10, 0.30], *P* = 0.33), whereas the TC levels of the AD group were significantly higher than that of the control group in Europe and the Americas (SMD: 0.29, 95% CI: [0.04, 0.53], *P* = 0.02) (the forest plot is reported in Figure 4). A significant association between the TC levels and AD also existed, but only when sample size was larger than 100 (SMD = 0.26, 95% CI: [0.07, 0.46], *P* = 0.007). When sample size was small (sample size < 100), there was no significant difference (SMD = −0.01, 95% CI: [−0.25, 0.24], *P* = 0.96; the forest plot is reported in Figure 5). It is reasonable to believe that data with a larger sample size are more trustworthy since a larger sample size in a clinical experiment is of great consequence. Finally, there was no strong correlation between TC levels and AD in both groups (SMD = 0.18, 95% CI: [−0.03, 0.38], *P* = 0.09 in “*before 2010”*; SMD = 0.16, 95% CI: [−0.05, 0.37], *P* = 0.14 in “*2010 and beyond,”* the forest plot is reported in Figure 6). Even with societal development and the changes in diet structure and living habits in the past decade, no significant changes appeared in the relationship between TC levels and AD.

**Table 4:** Results of stratified analysis.

**Subgroup**	**References**	**SMD**	**95% CI**	***P*-value**	**Effect model**	**Heterogeneity**	
						**I2 (%)**	***P*-value**
**TC**	Cankurtaran et al., 2005	0.17	[0.01, 0.32]	0.03	RE	82	<0.00001
Asia	Popp et al., 2012	0.1	[−0.10, 0.30]	0.33	RE	85	<0.00001
Europe and America	Alzheimer, 1907	0.29	[0.04, 0.53]	0.02	RE	71	0.0005
Mean age ≤ 70	Liu et al., 2019	0.45	[0.11, 0.79]	0.01	RE	86	<0.00001
Mean age > 70	Moher et al., 2009	0.05	[−0.09, 0.20]	0.47	RE	72	<0.00001
Sample size < 100	Anstey et al., 2017	−0.01	[−0.25, 0.24]	0.96	RE	60	0.006
Sample size ≥ 100	Strand et al., 2013	0.26	[0.07, 0.46]	0.007	RE	87	<0.00001
Before 2010	Wu et al., 2019	0.18	[−0.03, 0.38]	0.09	RE	59	0.009
2010 and beyond	Pappolla et al., 2003	0.16	[−0.05, 0.37]	0.14	RE	87	<0.00001
**HDL-C**	Moher et al., 2009	−0.15	[−0.34, 0.05]	0.15	RE	87	<0.00001
Mean age ≤ 70	Patterson, 2018	−0.50	[−0.76, −0.25]	0.0001	RE	60	<0.06
Mean age > 70	Strand et al., 2013	−0.03	[−0.26, 0.19]	0.78	RE	87	<0.00001
Sample size < 100	Dubois et al., 2014	−0.16	[−0.52, 0.21]	0.4	RE	68	0.008
Sample size ≥ 100	Rantanen et al., 2017	−0.12	[−0.34, 0.10]	0.25	RE	90	<0.00001
Before 2010	Liu et al., 2019	0.11	[−0.35, 0.58]	0.63	RE	92	<0.00001
2010 and beyond	Anstey et al., 2017	−0.3	[−0.45, −0.14]	0.0001	RE	65	0.002
**LDL-C**	Stroup et al., 2000	0.18	[−0.02, 0.38]	0.08	RE	87	<0.00001
Mean age ≤ 70	Patterson, 2018	0.59	[0.02, 1.16]	0.04	RE	92	<0.00001
Mean age > 70	Kivipelto et al., 2001	0.06	[−0.09, 0.22]	0.44	RE	70	<0.00001
Sample size < 100	Dubois et al., 2014	−0.06	[−0.26, 0.13]	0.53	RE	0	0.6
Sample size ≥ 100	Anstey et al., 2017	0.31	[0.05, 0.56]	0.02	RE	91	<0.00001
Before 2010	Liu et al., 2019	0.2	[−0.04, 0.43]	0.11	RE	70	0.002
2010 and beyond	Wu et al., 2019	0.17	[−0.13, 0.47]	0.27	RE	91	<0.00001
**TG**	Stroup et al., 2000	0	[−0.12, 0.12]	1	RE	62	0.0004
Mean age ≤ 70	Patterson, 2018	0.17	[−0.22, 0.55]	0.4	RE	83	0.0006
Mean age > 70	Kivipelto et al., 2001	−0.03	[−0.15, 0.09]	0.64	RE	51	0.02
Sample size < 100	Dubois et al., 2014	−0.13	[−0.56, 0.29]	0.54	RE	77	0.0006
Sample size ≥ 100	Anstey et al., 2017	0.04	[−0.07, 0.14]	0.51	RE	47	0.04
Before 2010	Dubois et al., 2014	−0.08	[−0.26, 0.10]	0.37	RE	39	0.14
2010 and beyond	Anstey et al., 2017	0.05	[−0.11, 0.21]	0.55	RE	69	0.0004

*SMD, mean difference; 95%CI, 95% confidence interval; RE, random effects model; TC, Total Cholesterol; TG, Triglyceride; HDL-C, High-density Lipoprotein Cholesterol; LDL-C = Low-density Lipoprotein Cholesterol*.

#### HDL-C and AD

Eighteen studies with 3,968 participants measured HDL-C levels; the meta-analysis results are presented in Table 3. These eighteen studies were all conducted in Asia. The pool effects indicated that HDL-C levels was not associated with AD (SMD = −0.15, 95% CI: [−0.34, 0.05], *P* = 0.15). With the high heterogeneity (*P* < 0.00001; *I*^2^ = 87%), the random-effects model was adopted. Figure 2B exhibits the forest plot of the pooled analysis.

**Figure 7:**
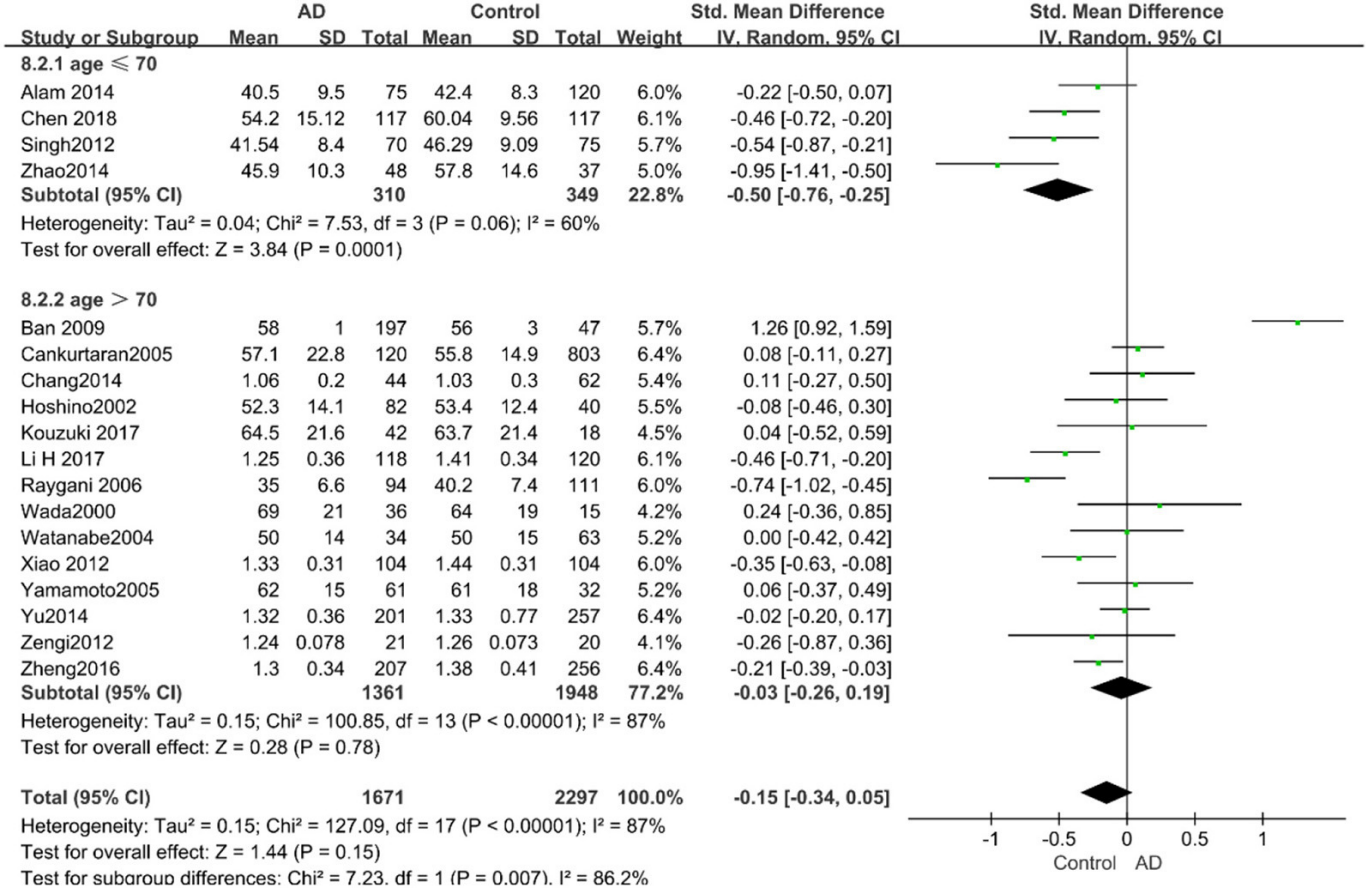
Forest plot of stratified analysis for the effects of HDL-C levels on AD risk in different age groups with a random-effects model. SMD, standardized mean difference; CI, confidence interval.

**Figure 8:**
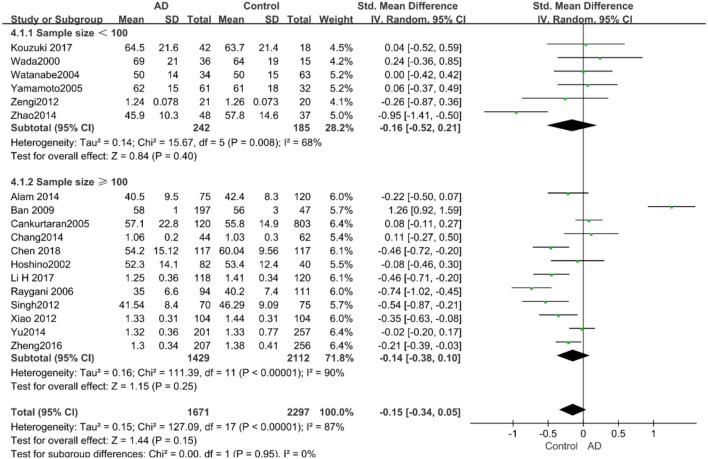
Forest plot of stratified analysis for the effects of HDL-C levels on AD risk in groups with different sample sizes with a random-effects model. SMD, standardized mean difference; CI, confidence interval.

**Figure 9:**
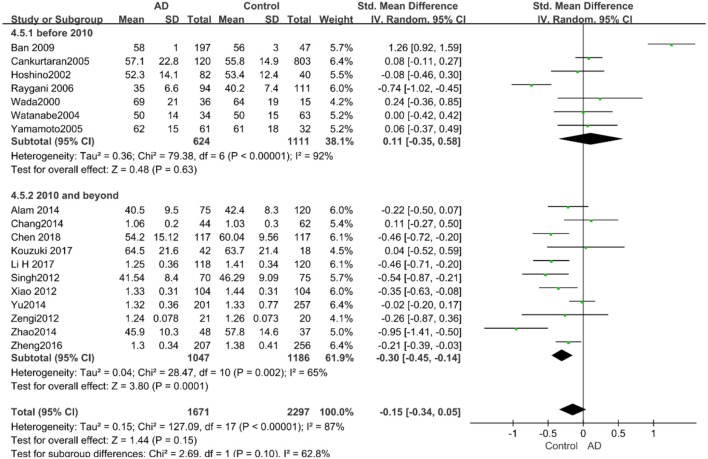
Forest plot of stratified analysis for the effects of HDL-C levels on AD risk in studies published before and after 2010 with a random analysis model. SMD, standardized mean difference; CI, confidence interval.

Similarly, after the stratified analysis, HDL-C levels were presented a negative association with AD in the “*mean age* ≤ *70 years old”* subgroup (SMD = −0.50, 95% CI: [−0.76, −0.25], *P* =0.0001). However, no obvious association was found between HDL-C levels and AD in the “*mean age* > *70 years old”* subgroup (SMD = −0.03, 95% CI: [−0.26, 0.19], *P* = 0.78; the forest plot is reported in Figure 7). There was no significant association in either the “*sample size* < *100”* (SMD = −0.16, 95% CI: [−0.52, 0.21], *P* = 0.40) or the “*Sample size* ≥ *100”* group (SMD = −0.14, 95% CI: [−0.38, 0.10], *P* = 0.25; the forest plot is reported in Figure 8). In the “*2010 and beyond”* subgroup, the AD group was significantly linked with decreased HDL-C levels (SMD = 0.11, 95% CI: [−0.35, 0.58], *P* = 0.0001), which was inconsistent with the result of the “*before 2010”* group (SMD = −0.30, 95% CI: [−0.45, −0.14], *P* = 0.63, the forest plot is reported in Figure 9). The results of the stratified analysis are shown in Table 4. HDL-C levels had a positive correlation with cognitive functioning in people under 70 years old. In addition, in line with TC levels, HDL-C levels had no strong relation with AD in the past decade.

#### LDL-C and AD

A total of 17 studies with 3,862 participants measured LDL-C levels and the results of the meta-analysis are listed in Table 3. These studies were all conducted in Asia. After pooling data, the results revealed that LDL-C levels had no significant relation with AD (SMD = 0.18, 95% CI: [−0.02, 0.38], *P* = 0.08). Figure 2C exhibits the forest plot of the pooled analysis.

**Figure 10:**
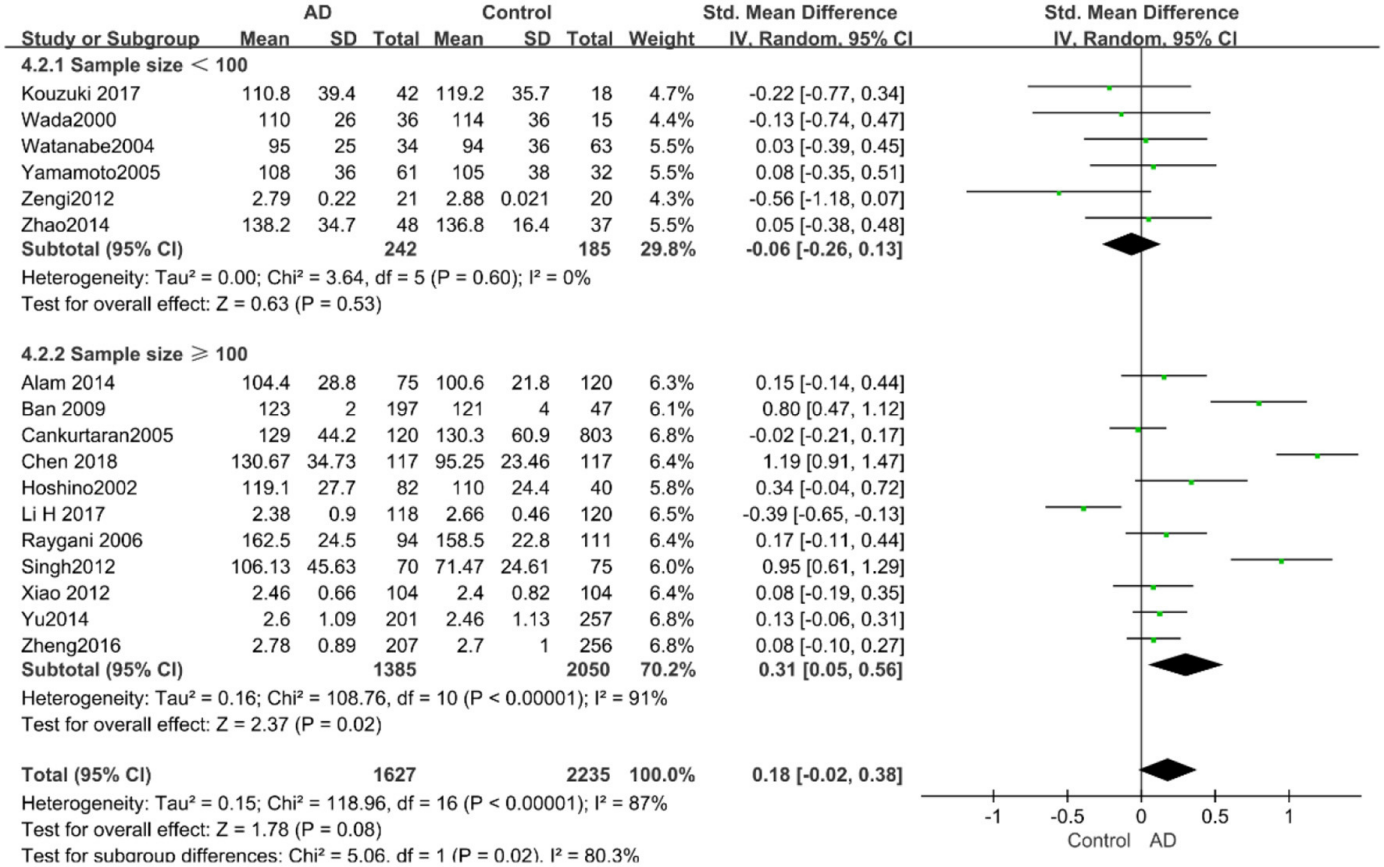
Forest plot of stratified analysis for the effect of LDL-C levels on AD risks in groups with different sample sizes with a random-effects model. SMD, standardized mean difference; CI, confidence interval.

**Figure 11:**
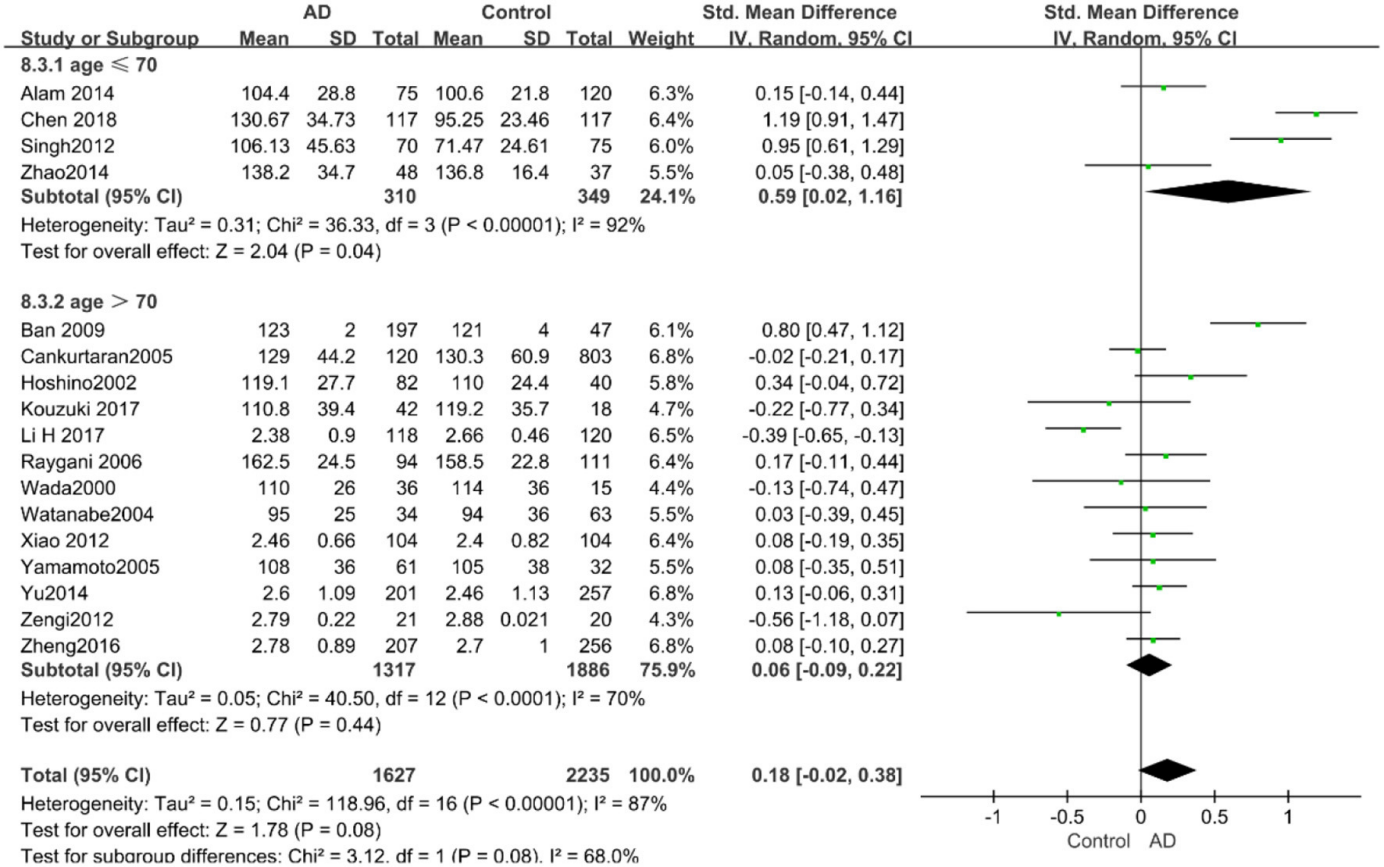
Forest plot of stratified analysis for the effects of LDL-C levels on AD risk in different age groups with a random-effects model. SMD, standardized mean difference; CI, confidence interval.

**Figure 12:**
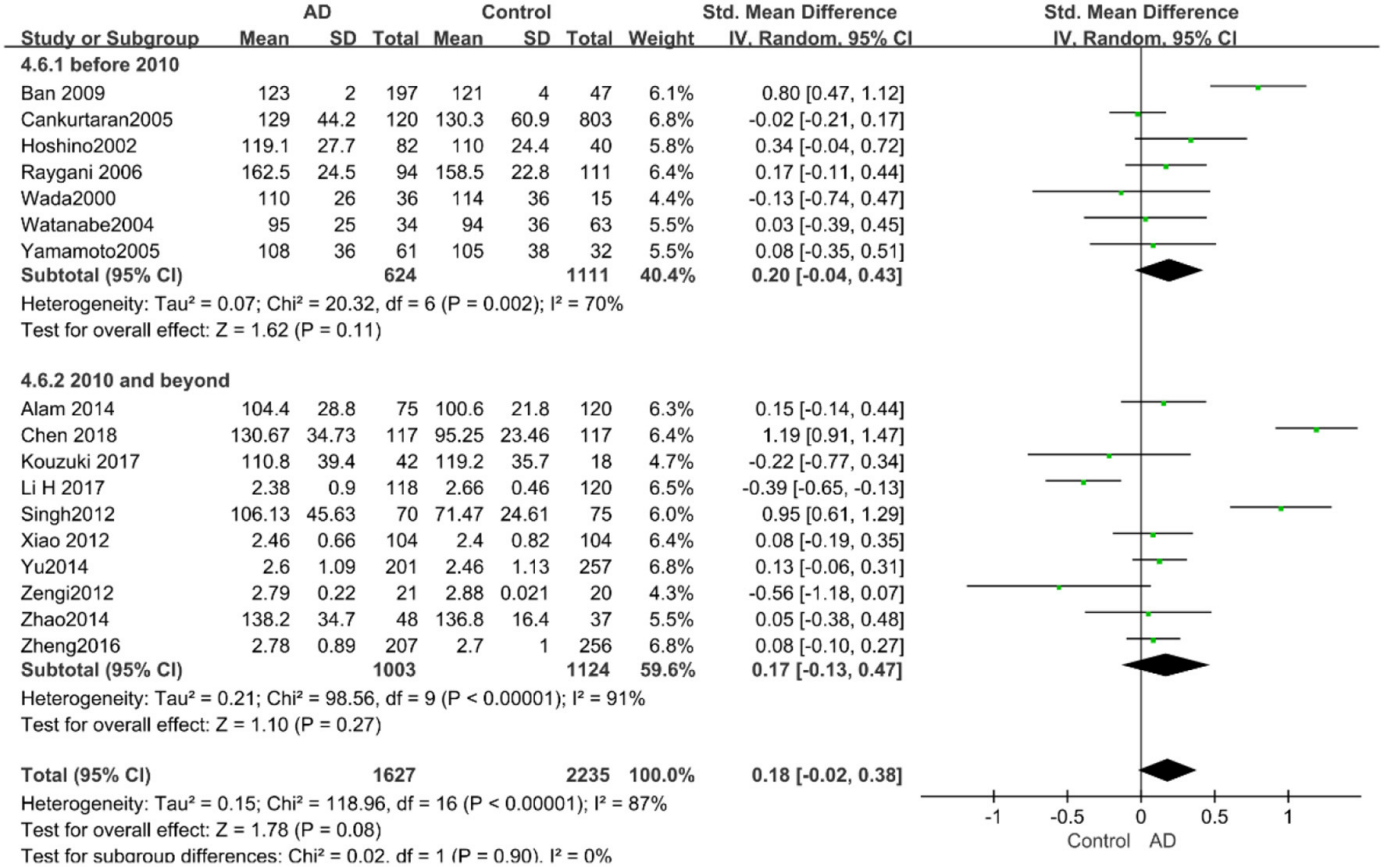
Forest plot of stratified analysis for the effects of LDL-C levels on AD risk in studies published before and after 2010 with a random-effects model. SMD, standardized mean difference; CI, confidence interval.

According to the results of the stratified analyses, in the group of “*sample size* ≥ *100,”* the AD group indicated significant correlation with increased LDL-C levels (SMD = 0.31, 95% CI: [0.05, 0.56], *P* = 0.02), whereas there was no strong association between LDL-C levels with AD in the “*sample size*< *100”* group (SMD = −0.06, 95% CI: [−0.26, 0.13], *P* = 0.53; the forest plot is reported in Figure 10). There was also no significant difference in the groups of “*mean age* ≤ *70 years old”* (SMD = 0.59, 95% CI: [0.02, 1.16], *P* = 0.04) and of “*mean age* > *70 years old”* (SMD = 0.06, 95% CI: [−0.09, 0.22], *P* = 0.44; the forest plot is reported in Figure 11). When studies were stratified according to the time of publication, no significant difference was found between the two subgroups of “*before 2010”* (SMD = 0.20, 95% CI: [−0.04, 0.43], *P* = 0.11) and “*2010 and beyond”* (SMD = 0.17, 95% CI: [−0.13, 0.47], *P* = 0.27; the forest plot is reported in Figure 12). As mentioned before, studies involving a larger sample size are more persuasive. In patients who were under 70 years old, there was a negative association between LDL-C levels and cognitive functioning. Therefore, we may find a new method to affect cognition in its early stages at the LDL-C level. Timing is important; after age 70, it may make no sense to slow the progression of AD through this method.

#### TG and AD

There were 17 studies covering 3746 participants that measured TG levels. The result of meta-analysis is exhibited in Table 3. These studies were all conducted in Asia. The results of the pooled effects showed no significant association between TG levels and AD (SMD = −0.00, 95% CI: [−0.12, 0.12], *P* = 1.00). Figure 2D indicates the forest plot of the pooled analysis.

**Figure 13:**
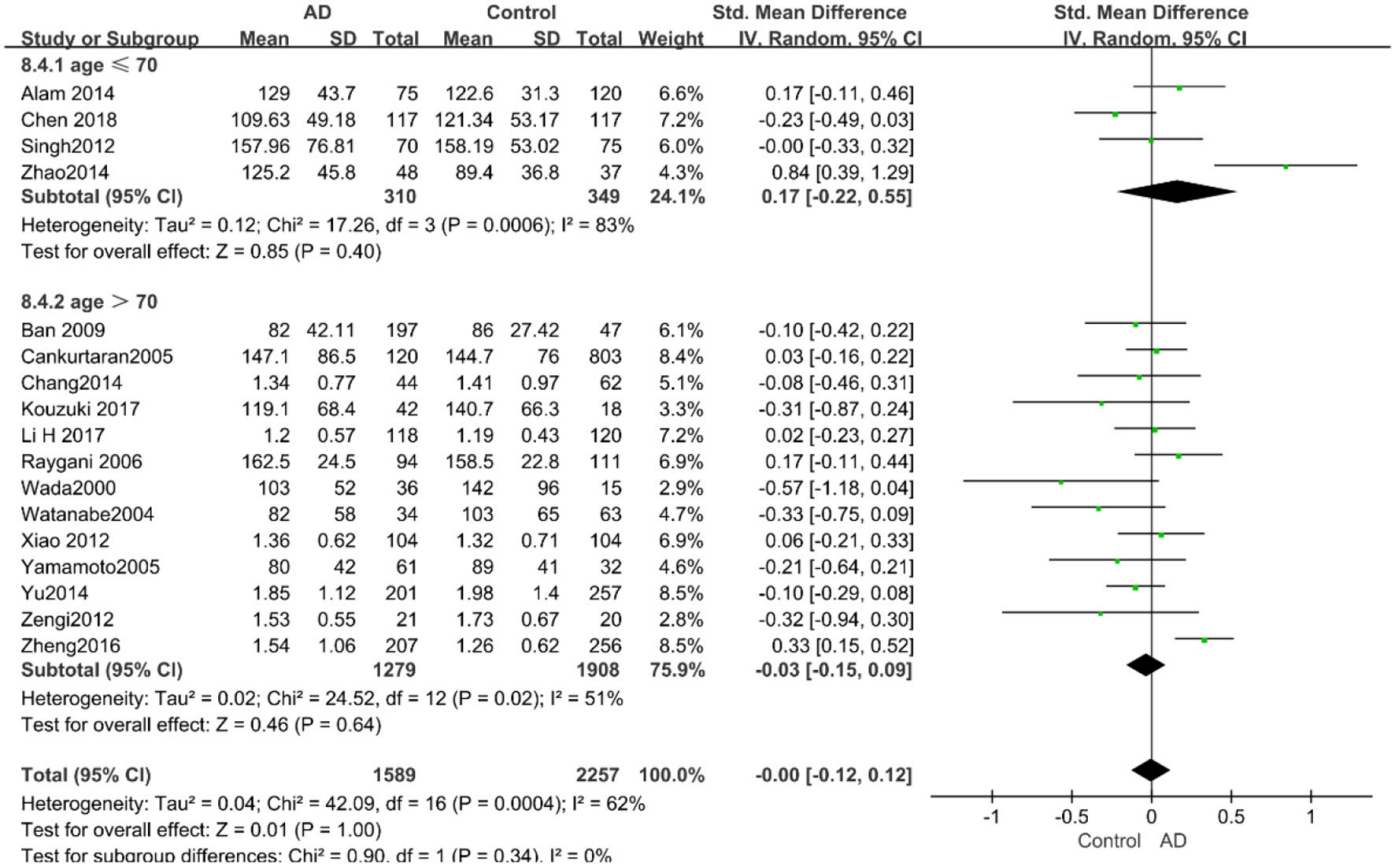
Forest plot of stratified analysis for the effects of TG levels on AD risk in different age groups with a random-effects model. SMD, standardized mean difference; CI, confidence interval.

**Figure 14:**
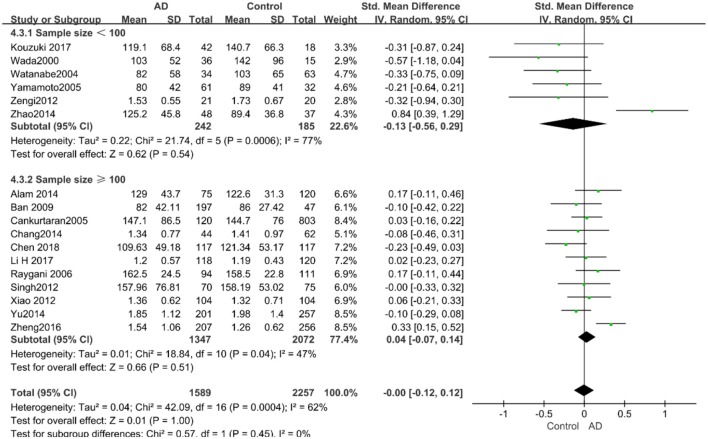
Forest plot of stratified analysis for the effects of TG levels on AD risk in groups with different sample sizes with a random-effects model. SMD, standardized mean difference; CI, confidence interval.

**Figure 15:**
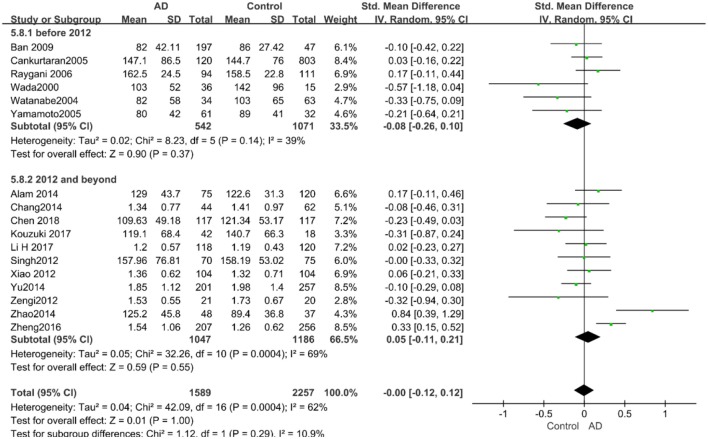
Forest plot of stratified analysis for the effects of TG levels on AD risk in studies published before and after 2010 with a random-effects model. SMD, standardized mean difference; CI, confidence interval.

The results of the pooled effects of all subgroups demonstrated no associations between TG and AD. The results were listed below: “*mean age* ≤ *70 years old”* (SMD = 0.17, 95% CI: [−0.22, 0.55], *P* = 0.40), “*mean age* > *70 years old”* (SMD = −0.03, 95% CI: [−0.15, 0.09], *P* = 0.64; the forest plot is reported in Figure 13), “*sample size*< *100”* (SMD = 0.13, 95% CI: [−0.56, 0.29], *P* = 0.54), “*Sample size* ≥ *100”* (SMD = 0.04, 95% CI: [−0.07, 0.14], *P* = 0.51; the forest plot reported in Figure 14). We have found no obvious association between the TG levels and AD in either the subgroups of “*before 2010”* (SMD = −0.08, 95% CI: [−0.26, 0.10], *P* = 0.37) or of “*2010 and beyond”* (SMD = 0.05, 95% CI: [−0.11, 0.21], *P* = 0.55; the forest plot is reported in Figure 15).

## Discussion

The aims of this study were to validate the association between AD and blood lipid levels, and whether blood lipid profiles could be used as biomarkers for AD diagnosis. In summary, our meta-analysis demonstrated five points: (a) elevated TC levels were associated with a higher risk of AD in later life, especially in Occidentals and subjects aged 60–70, which may be due to different eating habits in different regions, resulting in different cholesterol intakes (Menotti and Puddu, 2015). TC levels may be effective biomarkers for diagnosing AD. (b) In the subgroup of subjects aged 60-70, LDL-C levels were proven to have a positive correlation with AD, while HDL-C levels had a negative correlation with AD. (c) In the subgroup of studies containing a sample size over 100, LDL-C levels were found to have a positive correlation with AD in later-life. (d) The subgroup of studies published between 2010 and 2019 revealed that HDL-C levels had a negative correlation with AD in later-life. (e) TG levels had no significant association with AD in later-life.

Cholesterol is identified as the most plentiful type of lipid in the central nervous system, and ~25% of total amount of cholesterol is contained in human brain (Bjorkhem and Meaney, 2004; Cermenati et al., 2015), which is not only the primary component of lipid in nerve cell and glial cell membrane but also the key component of myelin sheath (Hussain et al., 2019). A recent study has found that microglia and the activation of NLRP3 inflammasome are vital for the pathogenesis of AD in tau, which supports the amyloid-cascade hypothesis of AD (Ising et al., 2019). Besides, cholesterol exerts an essential influence in plasma membrane regionalization, signal transduction, myelin sheath formation, and synaptic formation and maintenance (Chernick et al., 2019). Peripheral blood cholesterols can affect the human brain and AD through the blood-brain barrier (BBB) (Gamba et al., 2019). Our results showed that elevated TC levels were significantly associated with AD patients in later life. It can be inferred that elevated TC levels can be applied to diagnose AD and relative cognitive impairment in later-life patients. The result of a longitudinal study was consistent with our result that high TC levels were related to an increased risk of AD incidence (Ma et al., 2017). Additionally, some studies reported that hypercholesterolemia was identified as a risk factor for neurodegenerative disease, which resulted from the increase of the permeability of the BBB, inducing synaptic dysfunction and impairing neuron morphology (Merino-Serrais et al., 2019). Other studies have revealed that hypercholesterolemia predicted a progression to AD in patients with aMCI and that patients taking lipid-lowering drugs are less likely to develop AD (Li et al., 2011; Romero-Sevilla et al., 2018). Moreover, when cholesterol was lowered, the Precursor Protein of Aβ (APP) was swallowed into cells to decompose, which could reduce the extracellular presence of Aβ and the risk of AD (Martins et al., 2009). Furthermore, oxysterols (oxidative metabolites of cholesterols) may participate in AD progression via Liver X Receptors (LXR), which are key components in cholesterol homeostasis (Gamba et al., 2019; Merino-Serrais et al., 2019; Mouzat et al., 2019). For example, 24-hydroxycholesterol (24-OHC) can impede hyperphosphorylation of tau induced by deposition of Aβ in SK-N-BE cells by regulating the deacetylase sirtuin 1. As a neuroprotective oxysterol, 24-OHC was also validated to modulate synaptic function in hippocampal neurons of rats through LXR (Testa et al., 2018). Furthermore, genome-wide association studies (GWAS) verified that some genes like ABCA7, TREM2, DAPK1, and ADAMTS1 were associated with AD. The proteins expressed by these genes participated in tau binding proteins, endocytosis, innate immunity, APP, cholesterol metabolism, etc. (Kim et al., 2016; Efthymiou and Goate, 2017; Kunkle et al., 2019).

HDL-Cs are the most critical subtype of lipoprotein granules. Small HDL-C particles are able to enter into the brain, and they also circulate in the peripheral blood. As the only lipoprotein in CSF, HDL has been found to regulate intracellular cholesterol homeostasis and amyloid protein metabolism in the brain (Bahrami et al., 2019). In this study, we identified that HDL-C levels were negatively associated with AD, which is consistent with the previous finding that higher levels of HDL may reduce the risk of the late-onset AD (Reitz et al., 2010). This association may be due to the neuroprotective effect of small-sized HDL (Ohtani et al., 2018). HDL-C has also been verified to functionally prevent aggregation and polymerization of Aβ in the human brain and prevent the inflammation caused in neurodegenerative processes (Koudinov et al., 1998; Olesen and Dago, 2000; Cockerill et al., 2001). Moreover, HDL-C was able to change the APP degradation by interrupting the clearance of Aβ and promoting the amyloid fibrillary formation (Koch and Jensen, 2016). On the other hand, decreased HDL-C levels may reduce the risk of cardiovascular and cerebrovascular diseases (CVD) as well as the development of AD or vascular dementia. Meanwhile, HDL-C deficiency had been identified to contribute to cognitive impairment by affecting the risk of atherosclerosis (Bahrami et al., 2019).

LDL-C levels were presented significantly higher in AD patients than in elderly people with normal cognition in studies with a large sample size (over 100 participants). A possible explanation for the implications of our results is that LDL-C levels might promote the metabolism of Aβ in the human brain, the formation of cortical plaques and tangles, as well as the creation of neurotoxicity fibrils and neuritis, to speed up the progression of cognitive impairment related to dementia (Galasko et al., 1997; Gandy, 2005; Lv et al., 2016). In accordance with the results of the present study, a longitudinal study of elderly Chinese people has revealed that higher TC levels and LDL-C levels were associated with faster cognitive decline (Ma et al., 2017). Notably, as a key factor in cholesterol homeostasis, ApoE4 could decrease LDL receptor and LDL clearance, and increase cholesterol level by binding with TG-rich very-low-density lipoprotein. In addition, ApoE4 has been verified to contribute to the susceptibility for AD by disordering the lipids and cholesterol levels (Henry et al., 2018). Furthermore, LDL-C had been found positively associated with the densities of neuron plaques, which hints at the onset of the neurodegenerative disease (Lesser et al., 2011). However, higher LDL-C levels have also been reported to be positively associated with better memory function. It may be due to the fact that LDL-C may negatively be associated with AD only when the increase lasts for a long enough duration (Leritz et al., 2016). Therefore, follow-up cohort studies with a large sample size are needed to strengthen our results in this study.

We were not able to prove that TG levels had any association with AD in our study. However, previous studies reported a negative relationship between TG and memory performance (de Frias et al., 2007; Morley and Banks, 2010; van den Kommer et al., 2012; Leritz et al., 2016). It may be due to the fact that higher levels of TG contribute to reduced cognitive performance via CVD risk factors (Iturria-Medina et al., 2016). This inconsistency may be due to the age differences of participants in various studies and the differing methods of measurement.

Two specific biomarkers Aβ and Tau have been used to diagnose AD via amyloid imaging and monitoring their levels in CSF. However, only when cognitive symptoms developed apparently can they be applied clinically (Efthymiou and Goate, 2017). The present study investigated the possibility and feasibility of TCs as novel biomarkers of AD. Concerning the large number of studies and large sample sizes investigated in this study, the results could be promising. TC levels can be altered by several factors; in the future, lowering TC levels are expected to be an important factor in retarding or reversing the condition of cognitive decline in AD, and more RCT or cohort researches are necessarily needed to conduct. The highlights of this study are as follows: (a) a meta-analysis was conducted to assess the associations between blood lipid levels and AD and they were indeed linked. (b) Our results may be more representative and reliable since the population of our study was not limited to one nation or one continent. (c) The meta-analysis followed the MOOSE guidelines. Nevertheless, there are still several limitations we have to point out: (a) this study is a meta-analysis for case-control studies. The results will be more reliable if blood lipid levels are monitored over a long-term period in longitudinal researches. (b) The heterogeneity of our study appeared high even though we conducted stratified analyses. The source of high heterogeneity may result from varying stages of disease progression, multiple methods of blood lipid measurement, and other differences among demographic characteristics. (c) We may not control the confounding factors since some articles did not provide sufficient raw data about educational levels, the results of cognitive tests such as Mini-Mental State Examination (MMSE), methods of measurements, status of patients such as comorbidities, etc.

## Conclusion

In the present study, TC levels were strongly associated with AD in later-life. TC could be a new diagnostic biomarker of AD in later life. The significant associations between HDL-C/LDL-C and AD have not been discovered though, we may use them as references in a specific population. For example, lower HDL-C levels and higher LDL-C levels may relate to a higher risk of AD in populations aged 60–70. Remarkably, this kind of relationship should be explained carefully. TG levels are found to have no association with AD. Further comprehensive researches will be necessary in the future. As a biomarker, we are looking forward to prevent AD by monitoring the concentrations of TC. We also hope that it can be applied for early diagnosis of AD before the onset of symptoms and more AD patients benefit from it.

## Data Availability Statement

The raw data supporting the conclusions of this article will be made available by the authors, without undue reservation, to any qualified researcher.

## Author Contributions

QT, FW, JY, BL, and SW chose the topic. QT, JY, HP, and YL searched the literature. QT was responsible for literature searching, data analyzing, and writing of the first draft. QT, FW, and SW extracted and reviewed the data. SW and BL performed data management and figure modification. QT and BL modified the paper.

### Conflict of Interest

The authors declare that the research was conducted in the absence of any commercial or financial relationships that could be construed as a potential conflict of interest.
